# Recent Advances in Silver Nanowires Electrodes for Flexible Organic/Perovskite Light-Emitting Diodes

**DOI:** 10.3389/fchem.2022.864186

**Published:** 2022-03-10

**Authors:** Shuping Hou, Jie Liu, Feipeng Shi, Guo-Xu Zhao, Jia-Wei Tan, Gong Wang

**Affiliations:** ^1^ School of Information Engineering, Tianjin University of Commerce, Tianjin, China; ^2^ Center for Advanced Laser Technology, Hebei University of Technology, Tianjin, China; ^3^ Hebei Key Laboratory of Advanced Laser Technology and Equipment, Tianjin, China

**Keywords:** silver nanowires, organic light-emitting diodes, perovskite light-emitting diodes, flexible electronics, transparent conductive electrode

## Abstract

Flexible organic light-emitting diodes and perovskite light-emitting diodes (PeLEDs) have been investigated as an innovative category of revolutionary LED devices for next-generation flat display and lighting applications. A transparent conductive electrode is a key component in flexible OLEDs and PeLEDs, and has been the limitation of the development in this area. Silver nanowires (AgNWs) have been regarded as the most suitable alternative material in TCEs, due to the economical solution synthesis and compatibility with roll-to-roll technology. This mini-review addresses the advances in silver nanowires electrodes for flexible organic/perovskite light-emitting diodes, and the relationship between electrode optimization and device performance is demonstrated. Moreover, the potential strategies and perspectives for their further development of AgNWs-based flexible OLEDs and PeLEDs are presented.

## Introduction

Wearable electronics are revolutionizing how people interact with the world and each other (Afroj et al., 2020). Within the last 2 decades, the market trend of wearable electronics has stepped away from bulky, heavy, and wired electronics, and consumers are experiencing a world where devices are becoming smaller, lighter, and wirelessly connected with advanced smart technologies. (Han et al., 2019; Ma et al., 2020; Shi et al., 2021). Much of these changes were enabled by the successful development of novel electronic materials and miniaturization of electronic devices (N.Sgourou et al., 2017). To further enhance the functionality, practicality, and aesthetics of the next generation of wearable electronic products, soft and elastic flexible electronic products are needed. (Yuvaraja et al., 2020). In recent years, flexible organic light-emitting diodes (OLEDs) have become a promising technology for flat panel displays and solid-state lighting applications, thanks to their high efficiency, that could significantly reduce the energy consumption for lighting and information display (Xu et al., 2016; Lee J et al., 2017; Zou et al., 2020). Besides, metal halide perovskites (MHPs) are mechanically soft and process superior optoelectronic properties, and therefore show potential applications in next-generation wearable displays (Wang et al., 2020; Zhou et al., 2020).

Current OLEDs and PeLEDs rely heavily on the cost prohibitive component: ITO electrode (Khan et al., 2019). However, the ITO electrodes always suffer from degradation or failure if subjected to repeated bending, stretching, or other types of deformation in flexible electronics, which influence the efficiency and stability of the devices (S.Datta et al., 2020). Therefore, it is necessary to develop alternatives to ITO as the transparent conductive electrodes (TCEs) in flexible electronics (Yu et al., 2021). Silver nanowires (AgNWs) have been investigated as a potential ITO replacement, as the AgNW network can reproduce the high surface conductivity and visual transparency of ITO, and have the superior mechanical performance meanwhile (M.Abbasi et al., 2015; Tan et al., 2020). This mini-review focuses on the characteristics of AgNWs electrodes and their advantages overwhelming other flexible TCEs. Besides, the evolution of device performance of AgNWs-based flexible OLEDs and PeLEDs are summarized. Moreover, the future development of silver nanowires electrodes for flexible organic/perovskite light-emitting diodes is prospected.

### Silver Nanowires Electrodes

A transparent electrode is a key component in flexible/stretchable organic light-emitting diodes and perovskite light-emitting diodes (Zhu et al., 2019). Indium-tin oxide (ITO) has been more or less the only option for the transparent electrodes. ITO coated on glass is predominantly employed, whereas ITO coated on plastic substrates such as polyethylene terephthlate (PET) are used whenever flexibility is required (Lee S. M. et al., 2017). However, owing to the limited supply of indium, ITO becomes more and more expensive. In addition, ITO also has other inherent drawbacks due to the material’s properties, such as a lack of flexibility of the ITO layer, lack of chemical stability, fragility, and the toxicity of indium. All of the above-mentioned problems point to the urgent need to develop alternatives to ITO, especially for flexible/stretchable electronic devices (Sannicolo et al., 2016).

A number of alternative transparent electrodes have been investigated, the most notable ones include: 1) Conductive polymers represented by poly (3,4-ethylenedioxythiophene):poly (styrene sulfonate) (PEDOT:PSS). The conductivity of PEDOT:PSS can reach 1000 S/cm. PEDOT thin films can be transparent in the visible spectrum. However, PEDOT is unstable, the conductivity is still much lower than the ITO electrode, and the synthesis of the polymer is costly (Ma et al., 2021). 2) Carbon nanotubes (CNTs) appear to be a better solution because the conductivity of CNTs is higher than that of PEDOT. Ultrathin CNT coatings have been used to fabricate LEDs (He and Ye, 2015). However, CNTs are rather expensive and it is difficult to obtain uniform dispersions suitable for processing. The sheet resistance cannot be reduced to below 100 Ω/sq without significant loss of transparency. 3) Transparent conducting oxides other than ITO, such as alumina doped zinc oxide, gallium doped zinc oxide, etc. Aluminum doped zinc oxide is attractive as a less expensive and nontoxic alternative to ITO. However, the fabrication process still requires high vacuum physical sputtering or evaporation, and the coatings either do not have sufficiently low sheet resistance or low surface smoothness for processing into ultrathin organic films (Seok et al., 2019). 4) Graphene has also been investigated as transparent electrode. The best result is ∼30 Ω/sq at ∼90% transparency in which the graphene was synthesized through chemical vapor deposition and chemically p-doped by nitric acid. The stability of the doped electrode might be of concern due to the evaporation and migration of the nitric acid dopant (Liu et al., 2019). 5) Silver has a higher conductivity than conducting polymers, CNTs and doped ZnO. Based on calculations, the solar photon flux-weighted transmission versus sheet resistance for silver nanowires has a figure of merit exceeding 85% transmission at 10 Ω/sq sheet resistance versus 80% transmission for 10 Ω/sq ITO/glass substrate. Large-scale coating of silver nanowires has been demonstrated, and the transmittance at 550 nm can be over 80% (including the substrate) for 9 Ω/sq silver nanowire electrodes. Silver nanowires are highly attractive for transparent electrodes; however, improved processing techniques must be developed to form ultrathin coatings with low surface roughness (Guo et al., 2013).

Compared with other typical electrodes, the major advantages of silver nanowire transparent electrodes are the following: 1) The conductivity of silver is one to two orders of magnitude higher than ITO, doped ZnO, conducting polymers, and CNTs. An ultrathin coating of silver nanowires can form a highly conductive network. A high aspect ratio of nanowire length to diameter is essential for the network formation at low silver content per surface area. Besides, proper post-treatments are always used to improve the physical contact between adjacent AgNWs (Langley et al., 2013; Seo et al., 2021). 2) The polymer substrates for the silver nanowires are conventional polymers such as epoxies and polyacrylates, whose manufacturing cost is lower than that of glass. Furthermore, lamination is an effective approach to make the metal nanowires connect more closely with the polymer substrate and further reduce the roughness, which improve the quality of AgNWs (Kim et al., 2020; Kumar et al., 2021). 3) The silver nanowire/polymer substrate fabrication involves solution-based processing, which can be scaled up to roll-to-roll production for lowered cost. 4) The silver nanowire/polymer substrates are flexible and stable. Choosing the appropriate substrate polymer, one can fabricate highly flexible or even stretchable electronic devices under large deformations (Xiong et al., 2013). 5) The materials are nontoxic. The devices may be recycled or safely disposed to significantly reduce the overall environmental impact from electronic waste.

### Flexible Organic Light-Emitting Diodes

Flexible organic light-emitting diodes (FOLEDs) have been a promising technology for displays in wearable devices; however, the high manufacturing cost still hamper their large-scale applications (Li et al., 2013; Zhang et al., 2021). The manufacturing cost is mainly attributed to the high cost associated with substrate, electrode, and light-extraction structure (LES). AgNWs can be integrated in a streamlined solution-based process which is well-matched with Roll-to-Roll production for high throughput, which is an ideal approach to decrease the fabrication cost of FOLEDs. Besides, silver nanowires (AgNWs) have been investigated as a potential ITO replacement, as the AgNW network can reproduce the high surface conductivity and visual transparency of ITO (Zhang and Engholm, 2018).

In 2013, Gaynor et al. reported an ITO-free white OLED based on solution-processed AgNWs/PMMA electrodes, which showed a high power efficiency of 54 lm/W (Gaynor et al., 2013). In this work, the solution-possessed AgNW electrodes exhibited great advantages with commercial roll-to-roll production, demonstrating significant potentials to fabricate low-cost FOLED. Lee et al. developed a highly efficient OLED based on AgNW electrode, showing a low turn-on voltage of 3.6 V, a high current efficiency of 44.5 cd/A, and power efficiency of 35.8 lm/W (Lee et al., 2014). Chang et al. investigated a solution-processed s-MoO_x_-treated AgNW electrode with transmittance up to 95.9%, and low R_s_ of 29.8 Ω/sq (Chang et al., 2015). The resulting ITO-free FOLEDs exhibited a superior performance with power efficiency of 29.2 lm/W and EQE of 10.3%. Wei et al. constructed a highly conductive, smooth and transparent AgNW/PEDOT:PSS hybrid electrode with a resulting maximum current efficiency of 58.2 cd/A in 2017 (Wei et al., 2017).

### Flexible Perovskite Light-Emitting Diodes

Perovskites have been hotly pursued in recent years owing to their high photoluminescence quantum yield, long carrier diffusion length, strong light absorption, adjustable bandgap, and high carrier mobility (Song et al., 2019). After perovskite was first employed in light-emitting diodes, a slurry of research efforts has since been devoted in flexible PeLEDs (Kumawat et al., 2019). Flexible PeLEDs combine the excellent optoelectronic properties of perovskites with the potential of highly flexible electronics have come out as a novel category of revolutionary LED for panel displays and solid-state lighting applications (Lim et al., 2021; Rhee et al., 2021). To enable flexible applications of perovskite light-emitting diodes, flexible electrodes should satisfy the requirements of transparency, conductivity, and robustness. AgNWs electrode is an ideal choice for the flexible electrode and is widely applied in flexible and even stretchable PeLEDs. Besides, the perovskite active layer should feature good mechanical stability, and further engineering technology for perovskite film is important.

**FIGURE 1:**
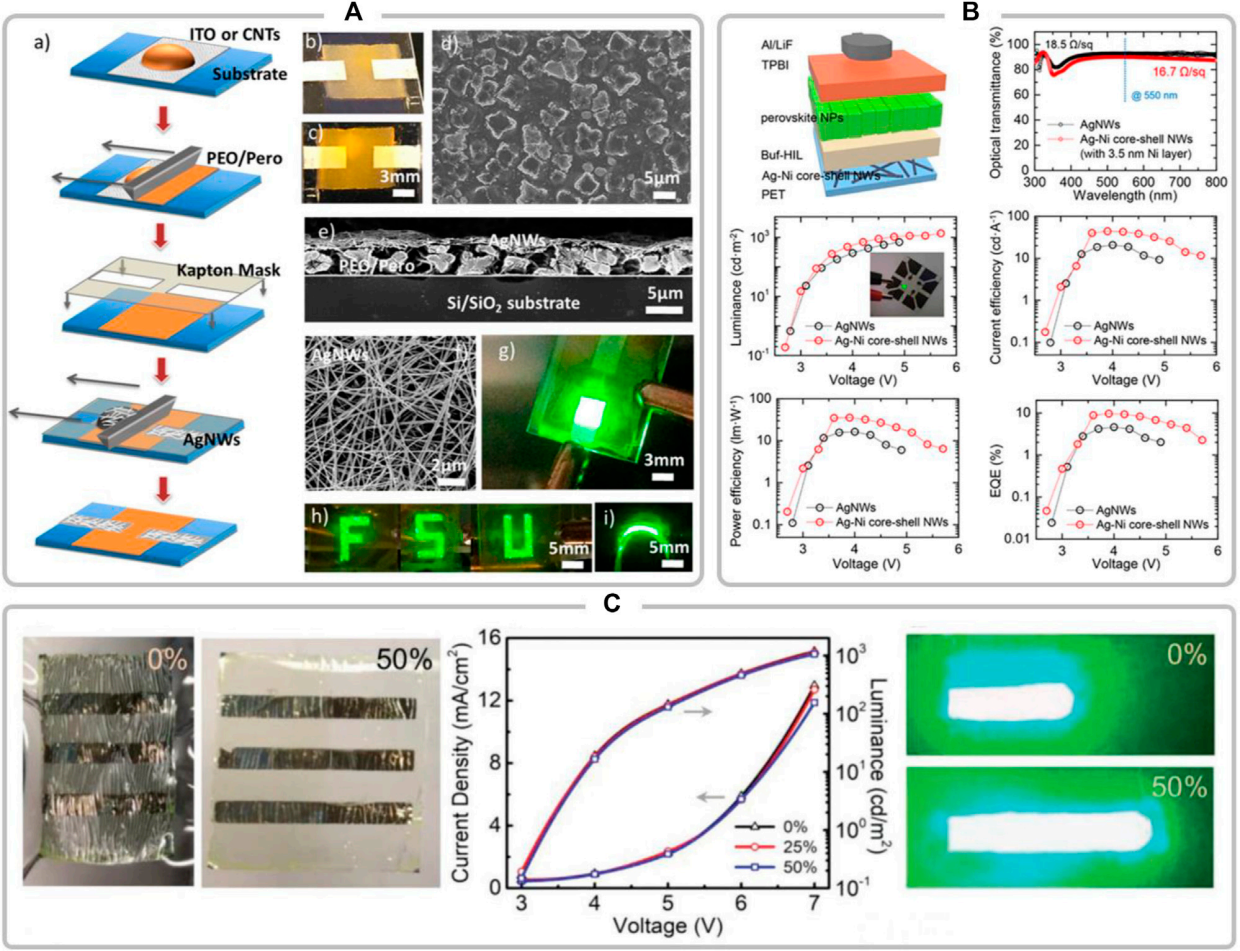
**(A)** Fully Printed Halide Perovskite Light-Emitting Diodes with Silver Nanowire Electrodes; reproduced with permission from R.Bade et al. (2016) **(B)** Electroplated Silver-Nickel Core-Shell Nanowire Network Electrodes for Highly Efficient Perovskite Nanoparticle Light-Emitting Diodes; reproduced with permission from Kang et al. (2020) **(C)** Stretchable Organometal-Halide-Perovskite Quantum-Dot Light-Emitting Diodes; reproduced with permission from Li et al. (2019).

As shown in Figure 1A, Bade et al. first reported the fully printed PeLEDs in 2016 with AgNWs as the cathode, and a printed composite film consisting of metal halide perovskites (MPHs) and poly (ethylene oxide) (PEO) as the emissive layer (R.Bade et al., 2016). The flexible PeLED can survive 5 mm radius of curvature without affection of the device performance. Subsequently, Chen et al. developed a morphology-controlled CsPbBr_3_-based PeLEDs fabricated on AgNWs/PET substrate in 2018 (Cheng et al., 2019). The flexible device showed a maximum current efficiency of 31.0 cd/A, and a maximum external quantum efficiency (EQE) of 10.1%. Later on, Kang et al. fabricated Ag-Ni core-shell nanowires by solution-electroplating to decrease the resistance, increase the work function of the electrodes, and prevent the reaction of AgNWs with the overlying perovskite nanoparticles (Kang et al., 2020). The PeLEDs based on Ag-Ni core-shell NW electrodes and FAPbBr_3_ nanoparticles exhibited an EQE of 9.67% (Figure 1B). Moreover, stretchable PeLEDs were also constructed on AgNWs/PI electrode with a luminescent efficiency up to 9.2 cd/A in Figure 1C (Li et al., 2019).

## Conclusion and Outlook

The cost-effective AgNWs-based OLEDs and PeLEDs take a big stride to the large-area and low-cost fabrication of flat displays in wearable devices. AgNWs with high aspect ratio process high transmittance and conductivity after proper post-treatments. Besides, lamination effectively make the close connect between AgNWs and polymer substrate and further reduce the roughness of electrode. Moreover, AgNWs/polymer substrate exhibit superior flexibility and stability even under repeated bending, enabling their applications in OLEDs and PeLEDs. As a result, the flexible AgNWs-based OLEDs and PeLEDs demonstrate good device performance and stability under deformations. Further efforts are also required to elucidate the physical mechanisms in flexible OLEDs and PeLEDs, especially when they are in bending condition. Furthermore, the encapsulation approach should be further discussed for their commercial applications.
